# Patient Perceptions of Dietary Supplement Use and Kidney Stone Disease

**DOI:** 10.3390/nu18101481

**Published:** 2026-05-07

**Authors:** David D. Kim, Megan L. Prochaska, Alex Weiss, Anna L. Zisman, Elaine M. Worcester, Luke F. Reynolds

**Affiliations:** 1 Section of Urology, Department of Surgery, The University of Chicago Medical Center, Chicago, IL 60637, USA; david.kim3@uchicagomedicine.org (D.D.K.); aweiss48@gmail.com (A.W.); 2 Section of Nephrology, Department of Internal Medicine, The University of Chicago Medical Center, Chicago, IL 60637, USA; mprocha2@bsd.uchicago.edu (M.L.P.); azisman@bsd.uchicago.edu (A.L.Z.); eworcest@uchicagomedicine.org (E.M.W.)

**Keywords:** kidney stones, apple cider vinegar, cranberry, turmeric, dietary supplements

## Abstract

Background/Objectives: There is a seemingly high prevalence of dietary supplement use in the kidney stone population. We aimed to understand patients’ perceptions of dietary supplements and their role in the management of kidney stones. Methods: We performed a standardized survey of patients presenting for management of kidney stones. We investigated the knowledge and use of apple cider vinegar (ACV), turmeric, and cranberry extract, as well as opinions on the safety of dietary supplements and sources of information. Descriptive statistics were used to summarize demographic and survey data. Results: Ninety-five patients were surveyed; 18 (18.9%) patients reported using ACV, 19 (20%) reported using cranberry extract, and 11 (11.6%) reported using turmeric. Similar numbers reported having heard of these dietary supplements being used for kidney stone prevention and/or treatment. Most patients believed these dietary supplements to be probably (ACV, *n* = 61, 64.2%; cranberry, *n* = 62, 65.3%; turmeric, *n* = 61, 64.2%) or definitely (ACV, *n* = 22, 23.2%; cranberry, *n* = 28; 29.5%; turmeric, *n* = 22, 23.2%) safe. For those who had heard about these supplements being used to treat or prevent kidney stones, friends/family (*n* = 25, 26.3%), online websites (*n* = 21, 22.1%), and social media (*n* = 15, 15.8%) were the most common sources of information. Conclusions: Apple cider vinegar, turmeric and cranberry extract have unknown risks or benefits in the management of kidney stones. Furthermore, their impact on stone pathophysiology remains unclear; however, many of our surveyed patient population uses them. Our study provides insight into patients’ use and perception of dietary supplements that clinicians should consider in the management of kidney stones. Further studies are needed to better counsel patients on the safety and efficacy of dietary supplements.

## 1. Introduction

Stone disease is a chronic medical condition that has been linked to several metabolic syndromes including obesity, diabetes mellitus, and hypertension [1]. It continues to be a common cause of morbidity with a prevalence of at least 10% in the United States (U.S.) [2]. It also continues to be increasingly burdensome on the U.S. healthcare system with costs estimated to be at least $2 billion a year [3]. Patients often initially present with symptoms, such as severe pain, that require surgical management, and stone disease is highly recurrent, with up to 39% of patients having symptomatic recurrence by 15 years [4]. Therefore, prevention remains an important part in the management of stone disease. Anecdotally, there is high rate of self-initiated use of non-prescription dietary supplements in our local patient population as part of a kidney stone management plan.

Diet is an important component of a kidney stone prevention plan. Several dietary factors have been associated with kidney stone prevention and risk. For example, a higher dietary intake of fruits, vegetables, and fluids (such as coffee, beer, and tea), and minerals such as calcium are associated with lower kidney stone risk, while a higher intake of animal protein has been associated with higher kidney stone risk [5,6,7]. Supplements have also been studied, albeit to a lesser degree. For example, calcium supplements and vitamin C supplements have both been associated with higher kidney stone risk [8,9]. Other supplements have been described as having protective properties related to antioxidant, antispasmodic, diuretic, and crystal inhibitory effects [10,11].

There is also a plethora of dietary supplements on the market claiming to treat or prevent kidney stones without data to confirm their benefits, and there is a lack of scientific evidence about the active ingredients in these supplements [12]. Recently, many patients in our local population have touted the protective and health-promoting benefits of ACV, turmeric, and cranberry extract. Given the anecdotal prevalence of the use of these three dietary supplements in our outpatient clinics, we sought to perform a pilot study to explore patients’ reported use and their perceptions on the role these dietary supplements play in the management of kidney stones.

## 2. Materials and Methods

### 2.1. Study Design and Population

This is a cross-sectional study of patients presenting to either a urologist or nephrologist for kidney stone care at a single institution between October 2024 and March 2025. Separate from their clinical care, study participants completed standardized surveys regarding basic kidney stone history and dietary supplement usage. The survey was developed with the support of the institution’s survey lab (Supplemental Material). Survey items included closed- and open-ended questions querying the knowledge and use of ACV, turmeric, and cranberry extract in the context of kidney stone disease. Questions also elicited patients’ opinions on the safety of these dietary supplements and sources of information. Surveys were only administered in English. Patient demographic information including age and sex was obtained through chart review; racial/ethnic identity and highest level of education completed were self-reported. Adults above the age of 18 years were included in the study. Surveys were administered during a visit to either a nephrologist’s or urologist’s outpatient clinic. All surveys were completed in person and in paper form before later being transcribed into an electronic database. Surveys were administered by an attending physician or research coordinator.

### 2.2. Statistical Analysis

Descriptive statistics were calculated to summarize demographic data and survey results. All analysis was carried out in SAS 9.4 (Cary, NC, USA). This study was approved by the institutional review board (IRB #24-1161).

## 3. Results

The patient demographic data are summarized in Table 1. Of the 100 patients surveyed, five patients self-reported multiple racial/ethnic identities, and so, were excluded from analysis. Of the remaining 95 patients, 36 (37.9%) were first-time stone formers and 59 (62.1%) were found to be recurrent stone formers. The median age of our cohort was 64 years. A total of 40 (42.1%) patients were male and 55 (57.9%) were female. A total of 40 (42.1%) patients identified as White, Caucasian, or European American and 45 (47.4%) identified as Black, African American, Black African, or Afro-Caribbean. Most patients held a Bachelor’s degree (4-year college degree) or higher.

The survey results are summarized in Table 2. Eighteen (18.9%) patients reported using ACV, 19 (20%) reported using cranberry extract, and 11 (11.6%) reported using turmeric. Similar numbers reported having heard of these dietary supplements being used for kidney stone prevention and/or treatment. While many patients (*n* = 39, 41.1%) reported not having heard of these supplements being used in the management of kidney stone disease, the majority believed ACV, cranberry, and turmeric to be probably (ACV, *n* = 61, 64.2%; cranberry, *n* = 62, 65.3%; turmeric, *n* = 61, 64.2%) or definitely (ACV, *n* = 22, 23.2%; cranberry, *n* = 28; 29.5%; turmeric, *n* = 22, 23.2%) safe. For those who had heard about these supplements being used to treat or prevent kidney stones, friends/family (*n* = 25, 26.3%), online websites (*n* = 21, 22.1%), and social media (*n* = 15, 15.8%) were the most common sources of information. Only 11 (11.6%) patients reported receiving information about these supplements from a physician or other healthcare professional.

## 4. Discussion

To better understand our local patient population’s use and perception of the safety profile of apple cider vinegar, cranberry, and turmeric, we performed a standardized survey of 100 patients presenting to either a urologist or nephrologist for consultation of kidney stone. Through our exploratory study, we found that many of our surveyed patients reported using any of the three dietary supplements and that most patients believed these dietary supplements to be probably or definitely safe.

Dietary recommendations remain a crucial component of kidney stone prevention and management. While several consumable items have been previously studied, including fruits, vegetables, fluids, calcium, and animal protein, little is known about the impact of certain common dietary supplements such as apple cider vinegar, turmeric, and cranberry extract. The mechanism underlying why apple cider vinegar may be helpful in kidney stone disease is not well understood. While apple cider vinegar does contain a high concentration of anion (acetate), the low pH of this dietary supplement limits its alkalinizing potential in urine. Still, it continues to be used as a popular dietary supplement [13].

Apple cider vinegar has been proposed to have several health-promoting benefits including improvements in fasting blood sugar, hemoglobin A1c, and antimicrobial properties [10,11,12,14,15]. In mice models, ACV has been shown to have a protective effect against oxidative injury in several cell lines while also lowering serum lipid levels [16]. Additionally, microbiological studies have demonstrated an antibacterial property of ACV against common antibiotic-resistant urinary tract pathogens [17]. However, its effects on the pathophysiology of kidney stone are not well understood.

Cranberry extract has also been found to be helpful in reducing the risk of symptomatic urinary tract infections (UTIs), but the existing data on its role in the prevention and treatment of kidney stones is conflicting [18,19,20,21]. Cranberry juice has been found to decrease urinary pH and urinary uric acid, while increasing the risk of calcium oxalate and uric acid stone formation [21,22,23]. This conflicts with another study which suggested that cranberry ingestion led to decreased oxalate and phosphate excretion with increased citrate excretion, leading to lower supersaturations of calcium oxalate [24].

Finally, there is a paucity of data on turmeric and its potential role in kidney stone pathophysiology. However, its high water-soluble oxalate content has been hypothesized to be lithogenic and ingestion in healthy individuals has been shown to result in higher urinary oxalate excretion at supplemental doses [25].

There is either a lack of supporting evidence or, at best, conflicting evidence that precludes clinicians from providing confident recommendations to their patients regarding ACV, cranberry, and turmeric supplement use with respect to kidney stones. Our study found that many patients are using them and that most of our patients believe these three dietary supplements to be probably or definitely safe. However, only 11% of patients reported obtaining information about these three dietary supplements from a physician or other healthcare professional. The widespread use of non-prescription dietary supplements is not limited to our local population, but present nationally [26]. Two prior studies investigated local patient populations’ knowledge and use of complementary alternative medicines (CAMs), including the three dietary supplements we investigated. Joshi et al. found that CAM use was common among kidney stone patients, especially in recurrent stone formers [27]. Green et al. also found that recurrent stone formers were more likely than first-time stone formers to have heard of and to have used CAMs [28]. Our patient demographic was markedly different from these two studies which had either a predominantly white male cohort or foreign-born Latinx ethnicity, respectively. About half of our survey participants identified as White, Caucasian, or European American and about another half identified as Black, African American, Black African, or Afro-Caribbean. The aforementioned studies also investigated a much broader range of products including herbal and natural remedies, fruits, and other commercially available anti-lithogenic products while our study focused on three commonly available over-the-counter supplements, which likely accounts for the lower reported use in our study (36% vs. 50% and 44%). Furthermore, our cohort was a well-educated population, with 63% of respondents holding a Bachelor’s (4-year college) degree or higher, which may have also differed from prior studies.

There are several important limitations to our study that must be considered. Firstly, the survey nature of our study gives rise to several biases including sampling and recall bias. Patients were recruited from a stone-specialty endourologist’s or nephrologist’s clinic, which may not necessarily reflect the general population. Furthermore, the highly educated cohort also may not necessarily reflect the general population, limiting external validity. Stone composition data were not collected, and therefore associations between dietary supplement use and types of kidney stones as well as the risk of recurrence cannot be inferred. Additionally, there is the potential for question bias despite our collaboration with survey specialists at our institution, which may have led to skewed or extreme answers. These three dietary supplements (ACV, turmeric, and cranberry extract) were chosen due to the anecdotal increase in patient interest. Other dietary and nutrient supplements are commonly used by patients in the general population but are outside the scope of this study as they were not specifically assessed. Finally, the relatively short (6-month) recruitment period introduces seasonal and temporal bias.

## 5. Conclusions

Apple cider vinegar, cranberry extract, and turmeric are supplements that have unknown risks and unknown benefits in the management of kidney stones, but are still used by over a third of our patient population. The majority of our cohort believed these to be probably or definitely safe. Our patients appear to obtain information on supplements from social media and friends/family. This data provides insight into patients’ use and perception of dietary supplements that clinicians should consider during the treatment and prevention of stone disease. Further studies are needed to better counsel patients with kidney stones on the safety and efficacy of supplement consumption. This will guide patient–provider discussions and shared decision making related to supplement use and kidney stone management.

## Figures and Tables

**Table 1:** Summary of patient demographics.

Characteristic	All Patients (*n* = 95)	First-Time Stone Former (*n* = 36)	Recurrent Stone Former (*n* = 59)
Patients, *n*	95	36	59
Age in Years, Median (Q1 ^a^, Q3 ^b^, Min, Max)	64 (46.8, 72.0, 21.4, 86.7)	60 (47, 71.5, 21.4, 85.5)	65 (46.9, 72.4, 28.5, 86.7)
Sex, *n* (%)			
Male	40 (42.1%)	15 (41.7%)	26 (44%)
Female	55 (57.9%)	21 (58.3%)	33 (56%)
Racial/Ethnic Identity, *n* (%)			
White, Caucasian, or European American	40 (42.1%)	10 (27.8%)	30 (50.8%)
Black, African American, Black African, or Afro-Caribbean	45 (47.4%)	22 (61.1%)	23 (39%)
Hispanic, Latin American, or Latina/Latino/Latinx	9 (9.5%)	4 (11.1%)	5 (8.5%)
Asian or Pacific Islander	1 (1.1%)	0 (0%)	1 (1.7%)
Level of School Completed, *n* (%)			
Did not complete Bachelor’s degree (4-year college degree)	36 (37.9%)	14 (38.9%)	22 (37.3%)
Bachelor’s degree (4-year college degree) or higher (Master’s, Doctorate or post-college professional degree)	59 (62.1%)	22 (61.1%)	37 (38.9%)
Patient Visit Type, *n* (%)			
New Patient	32 (33.7%)	19 (52.8%)	14 (23.7%)
Returning Patient	63 (66.3%)	17 (47.2%)	45 (76.3%)

^a^ First quartile, ^b^ Third quartile.

**Table 2:** Summary of survey results.

Question	All Patients (*n* = 95)	First-Time Stone Former (*n* = 36)	Recurrent Stone Former (*n* = 59)
Have you ever used …, *n* (%)			
ACV ^a^	18 (18.9%)	6 (16.7%)	12 (20.3%)
Cranberry	19 (20%)	6 (16.7%)	13 (22%)
Turmeric	11 (11.6%)	5 (13.9%)	6 (10.2%)
Have you heard of … used for stone prevention, *n* (%)			
ACV	20 (21.1%)	5 (13.9%)	15 (25.4%)
Cranberry	31 (32.6%)	7 (19.4%)	24 (40.7%)
Turmeric	12 (12.6%)	2 (5.6%)	10 (16.9%)
Have you heard of … used for stone treatment, *n* (%)			
ACV	13 (13.7%)	2 (5.6%)	11 (18.6%)
Cranberry	24 (25.3%)	8 (22.2%)	16 (27.1%)
Turmeric	8 (8.4%)	2 (5.6%)	6 (10.2%)
ACV is …, *n* (%)			
Definitely harmful	0 (0%)	0 (0%)	0 (0%)
Probably harmful	11 (11.6%)	2 (5.6%)	9 (15.3%)
Probably safe	61 (64.2%)	25 (69.4%)	36 (61%)
Definitely safe	22 (23.2%)	9 (25%)	13 (22%)
Cranberry is …, *n* (%)			
Definitely harmful	1 (1.1%)	0 (0%)	1 (1.7%)
Probably harmful	3 (3.2%)	1 (2.8%)	2 (3.4%)
Probably safe	62 (65.3%)	24 (66.7%)	38 (64.4%)
Definitely safe	28 (29.5%)	11 (30.6%)	17 (28.8%)
Turmeric is …, *n* (%)			
Definitely harmful	1 (1.1%)	0 (0%)	1 (1.7%)
Probably harmful	10 (10.5%)	2 (5.6%)	8 (13.6%)
Probably safe	61 (64.2%)	25 (69.4%)	36 (61%)
Definitely safe	22 (23.2%)	9 (25%)	13 (22%)
Source of Information, *n* (%)			
News (TV, radio, or online news)	4 (4.2%)	2 (5.6%)	2 (3.4%)
Social Media (TikTok, Facebook, YouTube, etc.)	15 (15.8%)	4 (11.1%)	11 (18.6%)
Online Website	21 (22.1%)	7 (19.4%)	14 (23.7%)
Doctor or other Healthcare Professional	11 (11.6%)	5 (13.9%)	6 (10.2%)
Friend or Family	25 (26.3%)	5 (13.9%)	20 (33.9%)
Other Source	4 (4.2%)	2 (5.6%)	2 (3.4%)
Have not heard about these supplements	39 (41.1%)	21 (54%)	20 (33.9%)

^a^ apple cider vinegar.

## Data Availability

The original contributions presented in this study are included in the article/Supplementary Material. Further inquiries can be directed to the corresponding author.

## Supplementary Materials

The following supporting information can be downloaded at: https://www.mdpi.com/article/10.3390/nu18101481/s1, Supplemental Material: Kidney stone and supplement use survey.

## Abbreviations

The following abbreviations are used in this manuscript:

ACV	Apple cider vinegar
U.S.	United States
UTI	Urinary tract infection
CAM	Complementary alternative medicine
