# Supplementary material for: Patient Perceptions of Dietary Supplement Use and Kidney Stone Disease
Source: Nutrients. 2026 May 7;18(10):1481. doi: 10.3390/nu18101481 (PMC13209987; doi:10.3390/nu18101481)
Supplement: Supplementary file 1 [file nutrients-18-01481-s001.zip › nutrients-4241696-supplementary.pdf]

Kidney Stone and Supplement Use Survey

The University of Chicago Sections of Urology and of Nephrology would like to know more about your use of <u>supplements related to kidney stones</u>. Your answers will help your doctor personalize your care. Additionally, your answers may be used in a research study, but ONLY if you provided consent to participate.

Please mark each answer with an X or check mark (✓ or X**).**

Q1. Have you used apple cider vinegar as a supplement?

- ○1 Yes → <u>Please answer Q1A, Q1B and Q1C</u>
- ○2 No

Q1A. Do you take apple cider vinegar as a pill or a liquid?

- ○1 Pill → How many pills at once? _________
- ○2 Liquid → How much at once? __________

Q1B. How often do you take apple cider vinegar?

_____ times per ______________

Q1C. About when did you start taking apple cider vinegar?

____ ○1days / ○2weeks / ○3months / ○4years ago

Q2. Have you used cranberry extract as a supplement?

- ○ Yes → <u>Please answer Q2A, Q2B and Q2C</u>
- ○ No

Q2A. Do you take cranberry extract as a pill or a liquid?

- ○1 Pill → How many pills at once? _________
- ○2 Liquid → How much at once? __________

Q2B. How often do you take cranberry extract?

_____ times per ______________

Q2C. About when did you start taking cranberry extract?

___ ○1days / ○2weeks / ○3months / ○4years ago

Q3. Have you used turmeric as a supplement?

- ○ Yes → <u>Please answer Q3A, Q3B and Q3C</u>
- ○ No

Q3A. Do you take turmeric as a pill or a liquid?

- ○1 Pill → How many pills at once? _________
- ○2 Liquid → How much at once? __________

Q3B. How often do you take turmeric ?

_____ times per ______________

Q3C. About when did you start taking turmeric?

___ ○1days / ○2weeks / ○3months / ○4years ago

Q4. Please list any other supplements you take:

____

____

The University of Chicago Sections of Urology and of Nephrology are conducting a study to better understand patients' views on supplements and their role in kidney stone disease. Your responses to the

following questions are OPTIONAL and results will only be used in aggregate. Please mark each answer with an X or check mark (✓ or X)**.**

Q5. Please select *ALL* that are part of your racial/ethnic identity:

- ☐1 White, Caucasian or European-American
- ☐2 Black, African-American, Black African, or Afro-Caribbean
- ☐3 Hispanic, Latin-American, or Latina / Latino / Latinx
- ☐4 Asian or Pacific Islander
- ☐5 Middle Eastern, North African, or Arab-American
- ☐6 Native American or Indigenous
- ☐7 Other, please list: ____

Q6. What is the *highest* level of school you have *completed*?

- ◯1 No formal schooling
- ◯2 Up to 8th grade
- ◯3 Some high school, no diploma and no GED
- ◯4 High school graduate, diploma or equivalent (for example., GED)
- ◯5 Some college, no degree
- ◯6 Trade, technical, or vocational school
- ◯7 Associate's degree (2-year college degree, AA or AS)
- ◯8 Bachelor's degree (4-year college degree, BA or BS)
- ◯9 Master's, Doctorate or post-college professional degree (MA, MS, MBA, PhD, MD, JD, etc.)

Q7. Please note whether or not you believe each of the following supplements is safe or harmful:

	Definitely harmful	Probably harmful	Probably safe	Definitely safe
Apple cider vinegar	◯1	◯2	◯3	◯4
Cranberry extract	◯1	◯2	◯3	◯4
Turmeric	◯1	◯2	◯3	◯4

Q8. For each of these supplements, please mark whether or not *you have heard or read about* them being used to prevent or treat kidney stones:

	Yes, heard of	No, *not* heard of
Apple cider vinegar used to *prevent* kidney stones	◯1	◯2
Apple cider vinegar used to *treat* kidney stones	◯1	◯2
Cranberry extract used to *prevent* kidney stones	◯1	◯2
Cranberry extract used to *treat* kidney stones	◯1	◯2
Turmeric used to *prevent* kidney stones	◯1	◯2
Turmeric used to *treat* kidney stones	◯1	◯2

Q9. Please select *ALL* sources from which you learned about any of these supplements as being useful to prevent or treat kidney stones:

- ☐1 News (TV, radio or online news report)
- ☐2 Social media (such as TikTok, Facebook, WhatsApp, YouTube, Instagram, or any other social media site)
- ☐3 Online website
- ☐4 Doctor or other healthcare professional
- ☐5 Friend or family
- ☐6 Somewhere else, please describe: ____
  - ◯99 Not applicable – I have not read or heard about any of these supplements.
